# Duloxetine hydrochloride loaded film forming dermal gel enriched with methylcobalamin and geranium oil attenuates paclitaxel-induced peripheral neuropathy in rats

**DOI:** 10.1016/j.ibror.2020.07.006

**Published:** 2020-07-15

**Authors:** Simerjeet Kaur Chahal, Rupinder Kaur Sodhi, Jitender Madan

**Affiliations:** a Department of Pharmacology, Chandigarh College of Pharmacy, Mohali, Punjab, India; b Department of Pharmaceutics, National Institute of Pharmaceutical Education and Research, Hyderabad, Telangana, India

**Keywords:** Chemotherapy-induced peripheral neuropathy, Film forming dermal gel, Paclitaxel, Duloxetine hydrochloride, Methylcobalamin

## Abstract

**Objective:**

In attempt to conquer the major concerns of oral duloxetine hydrochloride (like low bioavailability, intolerable side-effects and no regeneration of demyelinated nerve fibres) for the management of chemotherapy-induced peripheral neuropathy (CIPN), an alternative delivery of duloxetine hydrochloride was aimed for *in-vivo* optimization.

**Methods:**

A film forming dermal gel consisting of duloxetine hydrochloride was formulated and enriched with methylcobalamin and geranium oil. The formulated gel successfully qualified the various pharmaceutical characteristics of gel. Administration of paclitaxel (8 mg/kg/*i.p.* in four divided doses) for 4 alternate days induced the symptoms of peripheral neuropathy in rats. On 14th day, the responses to noxious stimulus (mechanical hyperalgesia, cold allodynia, and heat hyperalgesia) were increased and reached to its maximum. Thereafter, drug treatment with formulated dermal gel and oral duloxetine hydrochloride (30 mg/kg, once daily) was initiated for 2 weeks in different group of animals. On the 28th day animals were sacrificed to isolate sciatic nerve, to assess biochemical changes (TBARS, reduced GSH, total protein, TNF-α, IL-6) and for histopathological examinations of nerve sections using Hematoxylin-Eosin and Toludine blue staining methods.

**Results:**

Application of formulated dermal gel to paclitaxel-treated rats significantly improved paw-withdrawal latency responses during noxious stimulus testing, reduced the levels of TBARS, TNF-α, IL-6 and elevated the levels of reduced GSH as compared to paclitaxel treated rats. Histographs also indicated marked regeneration of the damaged nerve fibers. Topical delivery of duloxetine hydrochloride produced similar results in disparity to oral route. However, no significant disparity in responses was obtained with twice application of formulated dermal gel when compared to once daily application.

**Conclusion:**

Tremendous recovery from nociception, oxidation and inflammation in addition to nerve degeneration was achieved through dermal application of duloxetine hydrochloride in peripheral neuropathy.

## Introduction

Despite significant enhancement in cancer survival rate, chemotherapy-induced peripheral neuropathy (CIPN) is an emerging concern in cancer management with chemotherapeutic agents including taxanes and other agents (Staff et al., 2019). CIPN negatively impacts quality of life and causes immense debility in cancer patients and survivors (Farguhar-Smith and Brown, 2016). According o the latest cancer statistics, there are approximately 16 million cancer patients, which may be projected to 26.1 million by 2040 and almost 50–90 % of these patients receiving chemotherapy experience CIPN (Wolf, 2008). Clinically, CIPN presentation initiates in a “glove and stocking” distribution that may progress towards ankles and wrists from proximities to deficit sensory, motor and sometimes autonomic functions (Ferrier et al., 2013). Allodynia, hyperalgesia, tingling, burning, electric, stabbing, numbness, prickling sensations as well as changes in emotional, cognitive and social function often impose patient towards dose reduction or changeover to a different agent (Flatters and Bennett, 2006; Boland et al., 2010).

Paclitaxel (Taxol^Rx^) is a taxane-based drug commonly used for the treatment of various solid tumors such as breast, lung or ovarian cancer (Rivera and Cianfrocca, 2015; Dougherty et al., 2004). It has been suggested that paclitaxel increases stability of tubulin polymers that triggers microtubule disruption (Brzezinski, 2012; Carre et al., 2002), impedes axoplasmatic transport and promotes mitochondrial damage in A- and C-fibres (Velasco and Bruna, 2015) leading to wallerian degeneration in addition to distortion in activity and functions of Na+, K + and TRP ion channels (Salvemini et al., 2011; Xiao and Bennett, 2012; Conklin, 2004). These processes ultimately culminate into nociceptor sensitization and microtubule accumulation in Schwann cells. Moreover, segmental demyelination and activation of microglia in the Peripheral Nervous System (PNS) cross up the limits (Argyriou et al., 2008; U.S Pat Number WO2015089664A1).

Presently, numerous therapeutic interventions including antidepressants and antiepileptics are available for symptomatic relief (Hershman et al., 2014); however evidences for successful treatment of painful CIPN are limited (Pachman et al., 2014; Trivedi et al., 2015). Medications used to alleviate CIPN often lack efficacy. A randomized controlled study using duloxetine hydrochloride has been published for the treatment of CIPN (Hincker et al., 2019; Balayssac et al., 2011). Serotonin-norepinephrine reuptake inhibitor, duloxetine hydrochloride exhibits pain inhibitory action due to synergistic augmentation of both serotonergic and noradrenergic functions in the synaptic cleft. Duloxetine hydrochloride trims down the pain cascade from the periphery to the CNS (Smith et al., 2013; Zhou and Gebhart, 1997). Clinically prescribed oral duloxetine hydrochloride (30 mg, 60 mg and 120 mg once a day) has limited therapeutic efficacy in terms of intolerable side effects such as hepatic impairment, renal insufficiency, weight gain, anorexia, fatigue, dry mouth etc and poor nerve repair (Cruccu, 2007; Dworkin et al., 2007). Moreover, oral administration demonstrates poor bioavailability (≈50 %) due to first pass metabolism (Sharawy et al., 2017). Consequently, an alternative and more successful delivery may be a superior option that could ensures its safe use to manage CIPN symptoms.

Dermal drug delivery systems (DDDS) have been intensively considered in last decades as it delivers efficacious amount of drug directly into bloodstream across the skin (Rhee et al., 2008; El-Say et al., 2015). Furthermore, the film forming gel through DDDS provides better release of drugs in a controlled manner over a longer period of time in comparison to conventional methods of topical delivery (Ahmed and Khalid, 2014). Ideally, a drug having non-ionic behaviour with molecular weight (M_w_) less than 600 Da, log *P* value in the range of 1–4, solubility in both oils and water and low melting point (< 220 °C) penetrates the skin effortlessly (Singh and Bali, 2016). Evidences suggest that duloxetine hydrochloride possesses acceptable physicochemical and biopharmaceutical assets in terms of molecular weight (333.9 g/mol), log P (3.84), aqueous solubility (5 mg/mL) and melting point (162−162 °C) (Singh and Bali, 2016). Henceforth, in this study, film forming polymer-based duloxetine hydrochloride gel has been designed to fabricate. However, low permeability and solubility of duloxetine hydrochloride are the critical parameters. Utilization of permeation enhancers and dimethyl sulfoxide (DMSO) has been done for its adequate delivery through the skin. Moreover, it has been proposed that nurturing of formulation with nerve regeneration supplements like Methylcobalamin (active form of VitB_12_) and geranium oil (essential oil) could develop a more powerful solution to combat CIPN symptoms. Methylcobalamin plays a crucial role for biochemical metabolism, motor and sensory functions in nervous system (Sun et al., 2005). It serves as a precursor to synthesize lecithin, a major component of myelin sheath (Reyes-Garcia et al., 2004). It can speed up the myelination in the PNS and enhances peripheral nerve regeneration after injury in rats of the mammalian nervous system (Izumi and Kaji, 2007). Geranium oil is obtained from *Pelargonium graveolens* and reported for analgesic, anti-inflammatory and anti-oxidant activites (Frank et al., 2003). These nerve tonics help in rejuvenation of damaged myelin sheath and promote the growth at axonal level. The major objective of our study is to develop an efficient method that should not interfere with cancer treatment or block the activity of the oncolytic drugs and/or should not induce other side effects. Hence, the present study has been planned to formulate a duloxetine hydrochloride loaded film forming dermal gel to prevent paclitaxel-induced peripheral neuropathy.

## Materials and methods

### Chemicals and reagents

Paclitaxel injection was purchased from Bristol-Myers Squibb India Pvt. Ltd, Mumbai. Duloxetine hydrochloride and Methylcobalamin were procured in the pure powder form from the Sigma-Aldrich, USA. Carbopol ultrez, PEG 1500, HPMC, Propylene glycol, dimethyl sulfoxide and trichloroacetic acid were purchased from Merck, Germany. Glycerine and benzyl alcohol were procured from Thomas Baker Pvt Ltd, Mumbai, India. ELISA kits were purchased from PeproTech (Israel). Analytical grade reagents were used in this study and always freshly prepared before the use.

### Preparation of duloxetine hydrochloride loaded film forming dermal gel

Film forming dermal gel bearing duloxetine hydrochloride was formulated by cold dispersion method (Schmolka, 1972). In brief, carbopol ultrez (1.2 % w/w) was mixed in 50 mL of double distilled water (Table 1). Subsequently, PEG 1500 (0.4 % w/w) and HPMC (0.1 % w/w) were gently added in the mixture using homogenizer. Separately, methylcobalamin (0.1 % w/w) and duloxetine hydrochloride (1.5 % w/w) were dissolved in 10 mL double distilled water and DMSO (15 % w/w) respectively with continuous stirring and mixed in the above mixture with continuous stirring. Later on, propylene glycol (15 % w/w), glycerine (1 % w/w), geranium oil (1 % w/w) and benzyl alcohol (1.2 % w/w) were added with continuous stirring. Triethanolamine (0.5 % w/w) was incorporated to induce gelation and for maintaining pH of the gel (Srivastava et al., 2018). Double distilled water was added to adjust the final weight of the gel. Then gel was stirred on mechanical stirrer for 30 min to prevent entrapped air bubbles. The formulated dermal gel was stored in a wide mouthed bottle and preserved at cool temperature for further use.

**Table 1:** Composition of duloxetine hydrochloride loaded film forming dermal gel.

Sr.No.	Ingredients	Quantity (% w/w)
1	Carbopol ultrez	1.2
2	PEG 1500	0.4
3	HPMC	0.1
4	Propylene glycol	15
5	Duloxetine hydrochloride	1.5
6	Methylcobalamine (Vit B_12_)	0.1
7	Geranium oil	1
8	Glycerine	1
9	Benzyl alcohol	1.2
10	Dimethyl sulfoxide (DMSO)	15
11	Triethanolamine (TCA)	0.5
12	Double distilled water	63

### Characterization of duloxetine hydrochloride loaded film forming dermal gel

#### Appearance and homogeneity

The colour of formulated dermal gel was noticed and compared with marketed dermal gel. Moreover, homogeneity and consistency were tested visually and grittiness was examined through touch.

#### Measurement of pH

The pH of formulated dermal gel was measured accurately using a calibrated Digital pH meter (Hanna Instuments, Germany) (Liu et al., 2017). In brief, 1 g of gel sample was suspended in 50 mL of distilled water. Then, pH of formed homogenous dispersion was precise measured in triplicate (n = 3) and compared with marketed dermal gel.

#### Measurement of viscosity

The viscosity of formulated dermal gel was determined using LV-4 spindle equipped with rheometer (Brookfield DV-3 T + Rheometer) (Thakur et al., 2012). In brief, gel sample was added into the tube of rheometer and viscosity of the test sample was determined in triplicate (n = 3). The viscosity of marketed dermal gel was used for comparison.

#### Spreadability

Degree of area to which the dermal gel spreads at skin is called as spreadability. For its determination, 1 g of formulated dermal gel sample was spread on glass slide. Another glass slide was placed on first slide carefully to avoid any air entrapment and weight of 1000 g was put over them for 5 min. Later on, the upper glass slide was subjected to pull with the help of string (bearing 100 g weight at the other end) anchored to the hook. The time taken by upper glass slide to move a distance of 10 cm against the lower glass slide was noted in triplicate (n = 3). The formulations with short time intervals have better spreadability (Inoue et al., 2012).

Spreadability (S) can be calculated as:

S = M* L / T

Where, M depicts the mass (g) tied to upper glass slide, L is the length covered by glass slide and T denotes to time taken by glass slide to move.

#### Drug content

The quantitative extraction technique was used to determine drug content of formulated dermal gel. Accurately weighed 1 g of formulated dermal gel was dispersed in 50 mL of ethyl alcohol and gently heated for 15 min and filtered through 0.22-μm membrane filter (MDI, Ambala, India). The absorbance of the filtrate was measured at 290 nm (Kamila et al., 2007) using UV/Visible spectrophotometer (1800, Shimadzu, Kyoto, Japan). The drug content was estimated by using the following formula:

Drug content (g/g) = Actual drug content / Sample weight

### *In vivo* studies

#### Experimental animals

Wistar rats (180−200 g, either sex) were used for the *in vivo* evaluation of novel duloxetine hydrochloride loaded film forming dermal gel. Animals were acclimatized for one week to laboratory conditions prior to experimental study. The experimental protocol was duly approved by the Institutional Animal Ethics Committee (Reg. No. 1201/PO/Re/S/08/CPCSEA). The *in-vivo* study was carried out in accordance with the guidelines of the Committee for the Purpose of Control and Supervision of Experiments on Animals (CPCSEA).

#### Experimental protocol

Five different groups were used comprising of 6 rats in each group as articulated in Table 2. Paclitaxel was administered for 4 alternate days followed by drug treatments with formulated dermal gel (once a day and twice a day) and oral duloxetine hydrochloride (30 mg/kg, once a day) were initiated from day 14 to day 28. Based on experimental protocol (Table 2) various behavioural, biochemical and histopathological assessments were evaluated.

**Table 2:** Schematic representation of Experimental protocol with Groups and Treatment.

Groups and Treatment		
Sr. No	Group	Treatment
Group 1	Normal control	Rats were administered 0.9 % w/v normal saline (10 mL/kg; *p.o*.) for 28 days. The behavioural tests were performed on the different days, i.e., day 0, 2, 7, 14, 21 and 28. Thereafter, all the animals were sacrificed and sciatic nerves were isolated and subjected to biochemical analysis for the estimation of TBARS, reduced GSH, total protein, TNF-α, IL-6 and histopathological examinations.
Group 2	Paclitaxel control	Paclitaxel (8 mg/kg; *i.p*. in four divided doses) for 4 alternate days was administered to rats. The behavioural tests, the biochemical parameters and histopathogical examinations were assessed as mentioned in group 1.
Group 3	Paclitaxel + Oral Duloxetine HCl	Paclitaxel (8 mg/kg; *i.p.* in four divided doses) on days 1, 3, 5, and 7 was administered to rats followed by oral duloxetine HCl (30 mg/kg, once a day) initiated from day 14 to day 28. The behavioural tests, the biochemical parameters and histopathogical examinations were assessed as mentioned in group 1.
Group 4	Paclitaxel + Formulated dermal gel (once a day)	Paclitaxel (8 mg/kg; *i.p.* in four divided doses) on days 1, 3, 5, and 7 was administered to rats followed by application of formulated dermal gel (once a day) on hind paw initiated from day 14 to day 28. The behavioural tests, the biochemical parameters and histopathogical examinations were assessed as mentioned in group 1.
Group 5	Paclitaxel + Formulated dermal gel (twice a day)	Paclitaxel (8 mg/kg; *i.p.* in four divided doses) on days 1, 3, 5, and 7 was administered to rats followed by application of formulated dermal gel (twice a day) on hind paw initiated from day 14 to day 28. The behavioural tests, the biochemical parameters and histopathogical examinations were assessed as mentioned in group 1.

#### Generation of peripheral neuropathy

Paclitaxel (Taxol^Rx^) at the dose of 8 mg/kg was injected intraperitoneally in four divided doses, on days 1, 3, 5, and 7 to induce peripheral neuropathy in rats (Polomano et al., 2001).

#### Behavioural parameters

##### Mechanical hyperalgesia test

The mechanical nociceptive threshold is an index of mechanical hyperalgesia and is defined as the intolerable pressure or force (g) at which the rat withdraws its paw. Randall and Sellito method was used to measure mechanical nociceptive threshold (Randall and Selitto, 1957). In brief, pressure (g) was applied on the plantar surface of left hind paw and withdrawal of paw was noted to express the nociceptive threshold.

##### Cold allodynia test

Hypersensitivity to cold was assessed using the paw immersion test in cold water maintained at 10 °C. Left hind paw was immersed in cold water and maintained until the rats withdrew it from the water. The duration of paw immersion was recorded and presented as paw withdrawal latency in seconds for all the animals in each group. The cut-off time of 20 s was used (Kostich et al., 2016).

##### Heat hyperalgesia test

For the assessment of reactivity to noxious thermal stimuli, heat hyperalgesia of the hind paw was expressed using Eddy’s hot plate (Eddy et al., 1950). The rats were placed on the top of a controlled and maintained pre-heated hot plate (52.5 ± 0.5 °C). Withdrawal of hind paw was noted to assess the heat hyperalgesia with cut-off time set at 20 s.

#### Biochemical estimations

##### Collection of samples

Post drug treatment, cervical dislocation technique was used to sacrifice the rats. The sciatic nerves were isolated immediately and the uniform sciatic nerve homogenates were prepared with 0.1 M Sorenson phosphate buffer (10 %w/v, pH 7.4). The homogenates were placed in ice water for 0.5 h and centrifuged at 4 °C (10,000 rpm for 10 min). The supernatant of homogenates was separated and further used for biochemical estimations of sciatic nerve such as total protein content, thiobarbituric acid reactive substances level (TBARS), glutathione level (GSH), and levels of inflammatory mediators (TNF-α and IL-6). Some of the intact sciatic nerves were preserved in formalin for histopathological studies.

##### Estimation of total protein in sciatic nerve homogenate

Total protein content was calculated in the sciatic nerve homogenate by using bovine serum albumin (BSA) as a standard (Lowry et al., 1951). The absorbance was measured at 750 nm by using UV/Visible Spectrophotometer-1800, Shimadzu, Japan against suitably prepared blank. Total protein amount was expressed as mg/mL.

##### Estimation of thiobarbituric acid reactive substances (TBARS) level in sciatic nerve homogenate

An index of lipid peroxidation in terms of Thiobarbituric acid reactive substances (TBARS) was measured in the sciatic nerve (Niehaus and Samuelesson, 1968). Briefly, TBARS reagent was prepared by mixing equal volumes of thiobarbituric (0.37 %), trichloroacetic acid (15 %) and hydrochloric acid (0.25 N). 2 mL of TBARS reagent were mixed with 0.1 mL of sciatic nerve homogenate. The mixture was placed in water bath for 60 min for heating, cooled in ice bath for 10 min and then centrifuged for 10 min at 1000 rpm (4 °C). The absorbance of supernatant layer was measured at 535 nm using UV/Visible spectrophotometer against reference blank. TBARS level was calculated from the 1,1,3,3 - tetramethoxy propane standard curve. The values were expressed as nmol/mg of total protein.

##### Estimation of reduced glutathione (GSH) levels in sciatic nerve homogenate

Beutler’s method was used to assess reduced glutathione levels (Beutler et al., 1963). The equal volumes of sciatic nerve homogenate and trichloroacetic acid (10 %) were mixed and centrifuged at 1000 rpm for 10 min (4 °C). 0.5 mL of supernatant was mixed with 2 mL of 0.3 M disodium hydrogen phosphate buffer (pH 8.4). Then 0.25 mL of 0.001 M freshly prepared DTNB (5, 5´-dithiobis-2-nitrobenzoic acid) was added and absorbance of mixture was taken immediately at 412 nm using a spectrophotometer. Calibration curve was plotted using different concentrations of reduced glutathione (10−100 μm) and reduced glutathione were expressed as μmoles/mg of the total protein.

##### Estimation of inflammatory mediators (TNF-α and IL-6) in sciatic nerve homogenate

Levels of TNF-α and IL-6 were expressed in sciatic nerve homogenate using commercially available ELISA kits (Rat TNF-α Mini ELISA Development Kit, 900-M73 by PeproTech and Rat IL-6 Mini ELISA Development Kit, 900-M86 by PeproTech) as per manufacturer´s protocol. The colour development was monitored at 405 nm with wavelength correction set at 650 nm (Micro-Plate Reader, Cyberlab). The concentrations of TNF-α and IL-6 were measured in pg/mL.

##### Histopathological evaluation of sciatic nerve sections

Histopathological assessment of sciatic nerve was performed for estimation of nerve damage with the use of Hematoxylin and Eosin dye and Toluidine blue dye.

###### Hematoxylin and eosin staining method

The micrographs of the nerve sections stained with hematoxylin and eosin were captured with the support of light microscope (magnification × 100) (Sudoh et al., 2004).

###### Toluidine blue staining method

The micrographs of the toluidine stained nerve sections were also captured with light microscope (magnification × 100) (Ozaki et al., 2016).

#### Statistical analysis

The results were expressed as mean ± standard error of means (S.E.M.) and statistically analyzed by two-way analysis of variance (Bonferonni’s post hoc-test) using Graph Pad Prism Version-5.0 software. Biochemical results were statistically analyzed using one-way ANOVA (Tukey’s multiple range tests). The p-value < 0.05 was considered statistically significant.

## Results

### Formulated dermal gel exhibited desirable features for topical delivery

Film forming dermal gel bearing duloxetine hydrochloride, methylcobalamin and geranium oil was casted by “Cold dispersion” method using carbopol ultrez (gelling polymer), HPMC (film forming polymer), PEG1500 (plasticizer), propylene glycol (permeation enhancer), glycerine (humectant) and benzyl alcohol (preservative). The formulated dermal gel successfully qualified official and non official tests of dermal gel formulations. The colour of formulated dermal gel was observed to be pink (Table 3). In addition, the gel was homogenous in nature with absence of lumps. The pH of formulated dermal gel and marketed dermal gel was found in the range of 7.4 ± 0.3 and 7.5 ± 0.08, respectively with no significant difference (Unpaired t test, P > 0.05) (Table 3). The formulated dermal gel followed the Non- Newtonian flow. The spreadability of formulated dermal gel and marketed dermal gel was estimated to be 32 ± 0.14 g.cm/sec and 25 ± 0.10 g.cm/sec, respectively with significant difference (Unpaired t test, P < 0.05) (Table 3). Moreover, the drug content of formulated dermal gel was estimated and each g of gel was found to contain 0.012 ± 0.001 g of duloxetine hydrochloride (Table 3).

**Table 3:** Optimization of pharmaceutical characterization of duloxetine loaded film forming dermal gel in comparison to marketed gel.

Sr. No.	Pharmaceutical Characterisation of gel	Duloxetine loaded film forming dermal gel	Marketed gel
1	Colour	Pink	White
2	Homogeneity	Homogenous	Homogenous
3	pH	7.4 ± 0.3	7.5 ± 0.08
4	Viscosity	Non-Newtonian fluid	Non-Newtonian fluid
5	Spreadability (g.cm/sec)	32 ± 0.14	25 ± 0.10
6	Drug content	0.012 ± 0.001 g/g of gel	Nil

Values are expressed as mean ± standard error mean (S.E.M), n = 3.

### Formulated dermal gel revealed superior *anti-hyperalgesic* effect against mechanical hyperalgesia as compared to oral delivery of duloxetine hydrochloride

**Fig. 1:**
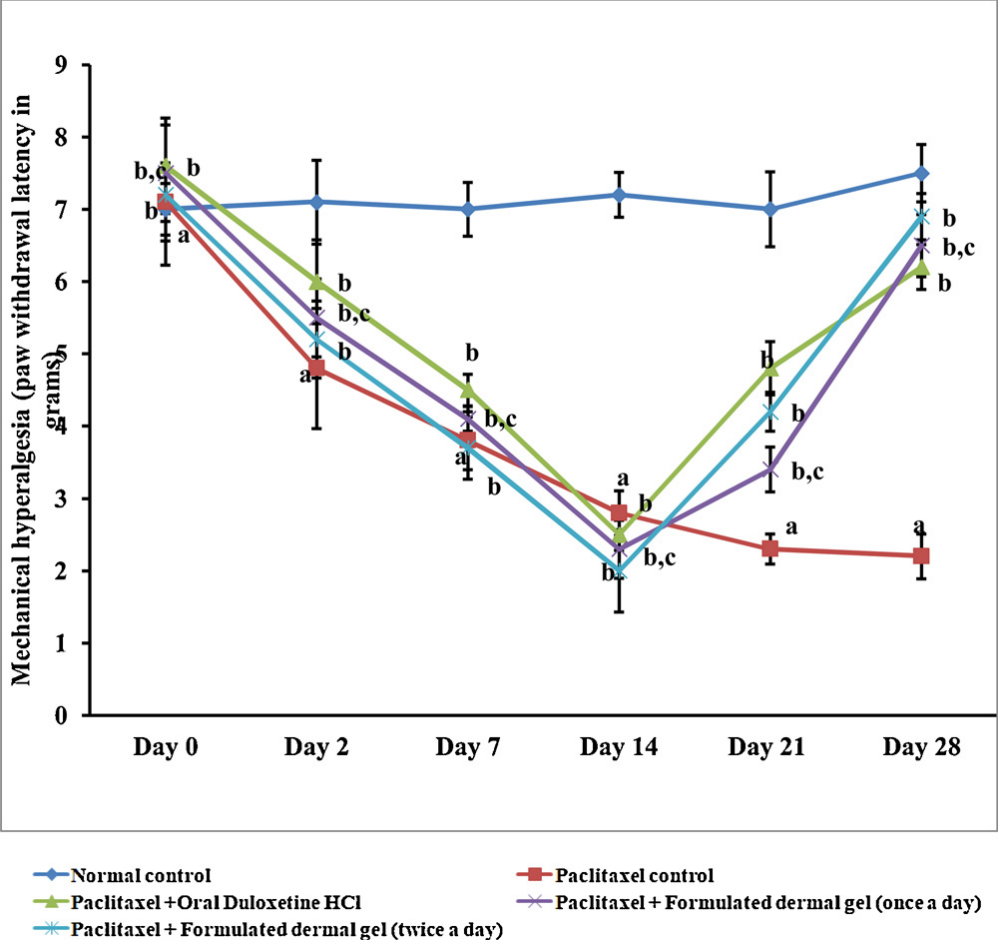
Graphical illustration of rat behavior modulation for mechanical hyperalgesia (paw withdrawal threshold in grams). Paw withdrawal latency test data for normal control, paclitaxel control, Paclitaxel + Oral Duloxetine HCl, Paclitaxel + Formulated dermal gel (once a day), Paclitaxel + Formulated dermal gel (twice a day) groups on different days of the investigation. **Normal control**: untreated group, **Paclitaxel control**: Paclitaxel (8 mg/kg; *i.p.* in four divided doses) for 4 alternate days, **Paclitaxel + Oral Duloxetine HCl**: Paclitaxel (8 mg/kg; *i.p.* in four divided doses) for 4 alternate days followed by duloxetine HCl (30 mg/kg, once a day for 14 days), **Paclitaxel + Formulated dermal gel (once a day)**: Paclitaxel (8 mg/kg; *i.p.* in four divided doses) for 4 alternate days followed by formulated dermal gel application once a day on hind paws of rats for 14 days, **Paclitaxel + Formulated dermal gel (twice a day)**: Paclitaxel (8 mg/kg; *i.p.* in four divided doses) for 4 alternate days followed by formulated dermal gel application twice a day on hind paws of rats for 14 days. The values are expressed as mean ± standard deviation (SD), n = 6. **a** depicts statistically significant differences (p < 0.05) between paclitaxel control and normal control. **b** depicts statistically significant differences (p < 0.05) between paclitaxel control vs Paclitaxel + Oral Duloxetine HCl/Paclitaxel + Formulated dermal gel (once a day)/Paclitaxel + Formulated dermal gel (twice a day). **c** depicts statistically insignificant differences (p > 0.05) between Paclitaxel + Formulated dermal gel (once a day) vs Paclitaxel + Oral Duloxetine HCl/Paclitaxel + Formulated dermal gel (twice a day).

Mechanical hyperalgesia has been assessed in the present study by pressure stimulation method (Randall and Sellito, 1957). Paclitaxel administration (8 mg/kg; *i.p.* in four divided doses) resulted in noteworthy development of mechanical hyperalgesia as indicated by significant (Two way ANOVA test, P < 0.05) reduction in hind paw withdrawal threshold (2.8 ± 0.31 g) in comparison to normal control rats (7.2 ± 0.31 g) (Fig. 1). Application of formulated dermal gel at hind paws of rats (once and twice a day for 2 weeks) significantly (One way ANOVA test, P < 0.05) prevented paclitaxel-induced decrease in the hind paw withdrawal threshold (6.5 ± 0.43 g) and (6.6 ± 0.32 g) respectively in comparison to paclitaxel treated rats (Fig. 1). However, no statistically significant variation in anti-hyperalgesic response was indicated with twice application in comparison to once daily application (Fig. 1). Similarly, oral administration of duloxetine hydrochloride (30 mg/kg, once a day for 2 weeks) to paclitaxel-treated rats significantly (Two way ANOVA test, P < 0.05) attenuated mechanical hyperalgesia (5.1 ± 0.31 g) when compared to paclitaxel control, however no significant variation was observed when oral group was compared to the formulated gel treated animals (Fig. 1).

### Formulated dermal gel exhibited better *anti-allodynic* potential against cold allodynia as compared to oral delivery of duloxetine hydrochloride

**Fig. 2:**
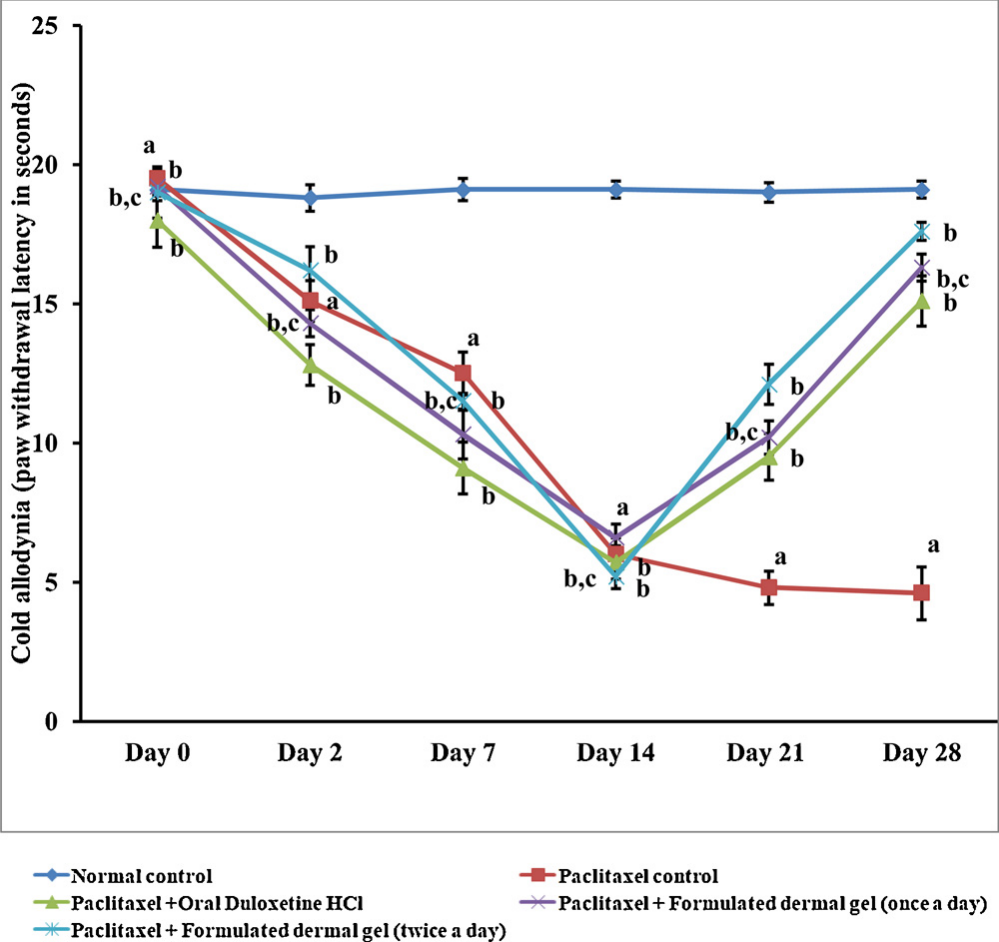
Graphical illustration of rat behavior modulation for cold allodynia (paw-lifting duration in seconds). Paw lifting latency test data for for normal control, paclitaxel control, Paclitaxel + Oral Duloxetine HCl, Paclitaxel + Formulated dermal gel (once a day), Paclitaxel + Formulated dermal gel (twice a day) groups on different days of the investigation. **Normal control**: untreated group, **Paclitaxel control**: Paclitaxel (8 mg/kg; *i.p.* in four divided doses) for 4 alternate days, **Paclitaxel + Oral Duloxetine HCl**: Paclitaxel (8 mg/kg; *i.p.* in four divided doses) for 4 alternate days followed by duloxetine HCl (30 mg/kg, once a day for 14 days), **Paclitaxel + Formulated dermal gel (once a day)**: Paclitaxel (8 mg/kg; *i.p.* in four divided doses) for 4 alternate days followed by formulated dermal gel application once a day on hind paws of rats for 14 days, **Paclitaxel + Formulated dermal gel (twice a day)**: Paclitaxel (8 mg/kg; *i.p.* in four divided doses) for 4 alternate days followed by formulated dermal gel application twice a day on hind paws of rats for 14 days. The values are expressed as mean ± standard deviation (SD), n = 6. **a** depicts statistically significant differences (p < 0.05) between paclitaxel control and normal control. **b** depicts statistically significant differences (p < 0.05) between paclitaxel control vs Paclitaxel + Oral Duloxetine HCl/Paclitaxel + Formulated dermal gel (once a day)/Paclitaxel + Formulated dermal gel (twice a day). **c** depicts statistically insignificant differences (p > 0.05) between Paclitaxel + Formulated dermal gel (once a day) vs Paclitaxel + Oral Duloxetine HCl/Paclitaxel + Formulated dermal gel (twice a day).

Administration of paclitaxel (8 mg/kg; *i.p.* in four divided doses) resulted in significant development of cold allodynia (6.0 ± 0.60 s), which was reflected as significant (Two way ANOVA test, P < 0.05) decrease in paw-lifting duration with cold water (10 °C) as compared to normal control rats (19.1 ± 0.30 s) (Fig. 2). Application of formulated dermal gel at hind paws of rats once daily (16.3 ± 0.49 s) and twice daily (17.6 ± 0.33 s) significantly (One way ANOVA test, P < 0.05) decreased paw-lifting duration (Fig. 2) as compared to paclitaxel-treated group. However, twice daily application of formulated dermal gel did not evident any statistically significant variation in anti-allodynic action when compared to once daily application of formulated dermal gel (Fig. 2). The oral administration of duloxetine hydrochloride (30 mg/kg, once a day for 2 weeks) indicated a significant (Two way ANOVA test, P < 0.05) improvement in paw-lifting duration (15.1 ± 0.90 s) as compared to paclitaxel treated group (Fig. 2).

### Formulated dermal gel demonstrated improved *anti-hyperalgesic* action against heat hyperalgesia when compared to oral administration of duloxetine hydrochloride

**Fig. 3:**
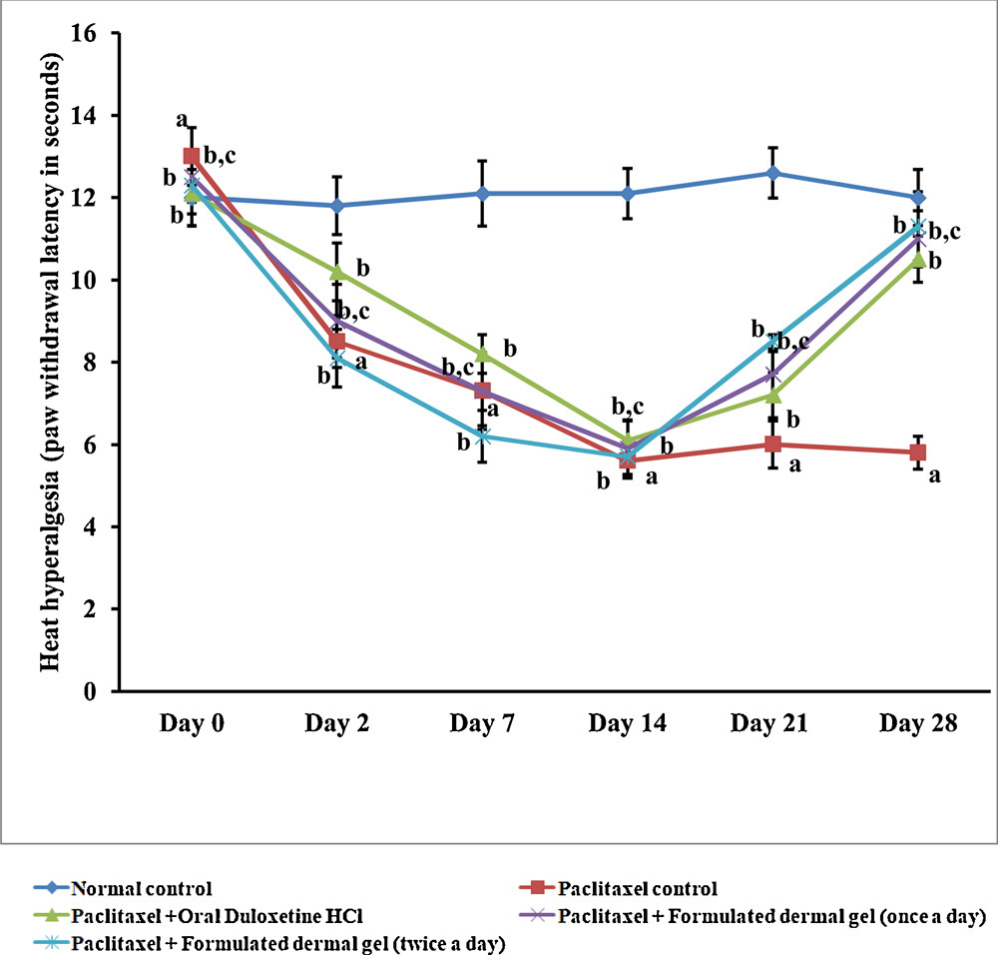
Graphical illustration of rat behavior modulation for heat hyperalgesia (paw withdrawal threshold in seconds). Paw withdrawal latency test data for for normal control, paclitaxel control, Paclitaxel + Oral Duloxetine HCl, Paclitaxel + Formulated dermal gel (once a day), Paclitaxel + Formulated dermal gel (twice a day) groups on different days of the investigation. **Normal control**: untreated group, **Paclitaxel control**: Paclitaxel (8 mg/kg; *i.p.* in four divided doses) for 4 alternate days, **Paclitaxel + Oral Duloxetine HCl**: Paclitaxel (8 mg/kg; *i.p.* in four divided doses) for 4 alternate days followed by duloxetine HCl (30 mg/kg, once a day for 14 days), **Paclitaxel + Formulated dermal gel (once a day)**: Paclitaxel (8 mg/kg; *i.p.* in four divided doses) for 4 alternate days followed by formulated dermal gel application once a day on hind paws of rats for 14 days, **Paclitaxel + Formulated dermal gel (twice a day)**: Paclitaxel (8 mg/kg; *i.p.* in four divided doses) for 4 alternate days followed by formulated dermal gel application twice a day on hind paws of rats for 14 days. The values are expressed as mean ± standard deviation (SD), n = 6. **a** depicts statistically significant differences (p < 0.05) between paclitaxel control and normal control. **b** depicts statistically significant differences (p < 0.05) between paclitaxel control vs Paclitaxel + Oral Duloxetine HCl/Paclitaxel + Formulated dermal gel (once a day)/Paclitaxel + Formulated dermal gel (twice a day). **c** depicts statistically insignificant differences (p > 0.05) between Paclitaxel + Formulated dermal gel (once a day) vs Paclitaxel + Oral Duloxetine HCl/Paclitaxel + Formulated dermal gel (twice a day).

Animals treated with paclitaxel (8 mg/kg; *i.p.* in four divided doses) showed significant (Two way ANOVA test, P < 0.05) heat hyperalgesia response (5.6 ± 0.42 s) as indicated by decrease in hind paw withdrawal threshold on Eddy^´^s hot plate test in comparison to response exhibited by normal control rats (12.1 ± 0.61 s) (Fig. 3). Application of formulated dermal gel at hind paws of paclitaxel-treated rats once daily (11.0 ± 0.68 s) and twice daily (11.3 ± 0.84 s) significantly (One way ANOVA test, P < 0.05) improved paclitaxel-induced decrease in the hind paw withdrawal threshold in comparison to paclitaxel-treated group (Fig. 3). Conversely, twice daily application of gel was inept to produce any statistically (Two way ANOVA test, P > 0.05) significant distinction of anti-hyperalgesic effect as compared to once daily treatment with formulated dermal gel (Fig. 3). Oral administration of duloxetine hydrochloride (30 mg/kg, once a day for 2 weeks) also reversed paclitaxel-induced heat hyperalgesia with a significant (Two way ANOVA test, P < 0.05) change in response (10.5 ± 0.56 s) when compared with paclitaxel-treated rats (Fig. 3). However, oral route did not exhibit any statistically considerable difference in comparison to topical treatment with duloxetine hydrochloride (Fig. 3).

### Anti-oxidant activity of duloxetine hydrochloride loaded film forming dermal gel on sciatic nerve

**Fig. 4:**
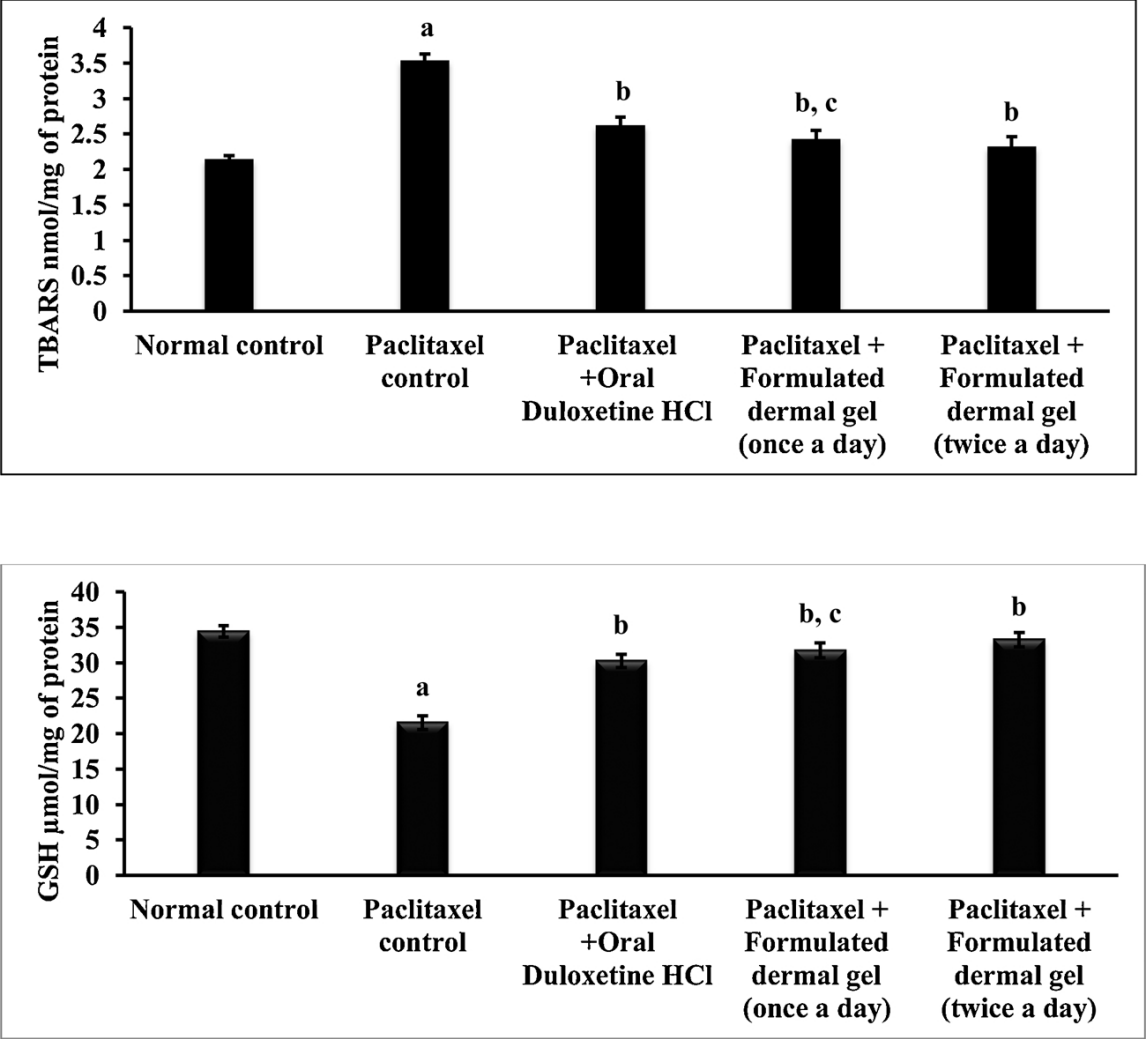
Measurement of TBARS level (nM/mg of protein) and reduced GSH level (μM/mg of protein) in rat sciatic nerve in normal control, paclitaxel control, Paclitaxel + Oral Duloxetine HCl, Paclitaxel + Formulated dermal gel (once a day), Paclitaxel + Formulated dermal gel (twice a day) groups. **Normal control**: untreated group, **Paclitaxel control**: Paclitaxel (8 mg/kg; *i.p.* in four divided doses) for 4 alternate days, **Paclitaxel + Oral Duloxetine HCl**: Paclitaxel (8 mg/kg; *i.p.* in four divided doses) for 4 alternate days followed by duloxetine HCl (30 mg/kg, once a day for 14 days), **Paclitaxel + Formulated dermal gel (once a day)**: Paclitaxel (8 mg/kg; *i.p.* in four divided doses) for 4 alternate days followed by formulated dermal gel application once a day on hind paws of rats for 14 days, **Paclitaxel + Formulated dermal gel (twice a day)**: Paclitaxel (8 mg/kg; *i.p.* in four divided doses) for 4 alternate days followed by formulated dermal gel application twice a day on hind paws of rats for 14 days. The values are expressed as mean ± standard deviation (SD), n = 6. **a** depicts statistically significant differences (p < 0.05) between paclitaxel control and normal control. **b** depicts statistically significant differences (p < 0.05) between paclitaxel control vs Paclitaxel + Oral Duloxetine HCl/Paclitaxel + Formulated dermal gel (once a day)/Paclitaxel + Formulated dermal gel (twice a day). **c** depicts statistically insignificant differences (p > 0.05) between Paclitaxel + Formulated dermal gel (once a day) vs Paclitaxel + Oral Duloxetine HCl/Paclitaxel + Formulated dermal gel (twice a day).

Paclitaxel (8 mg/kg; *i.p.* in four divided doses) administration for 4 alternate days significantly (Unpaired t test, P < 0.05) enhanced oxidative stress in the sciatic nerve measured as TBARS level (3.542 ± 0.09 nM/mg of protein) and reduced GSH level (21.58 ± 0.977 μM/mg of protein) in comparison to TBARS level (2.146 ± 0.05 nM/mg of protein) and reduced GSH level (34.42 ± 0.804 μM/mg of protein) of normal control animals (Fig. 4). Hind paw application of formulated dermal gel in rats for 2 weeks significantly (One way ANOVA test, P < 0.05) attenuated paclitaxel-induced oxidative stress in terms of decreased TBARS level (once daily: 2.431 ± 0.12 nM/mg of protein and twice daily: 2.327 ± 0.14 nM/mg of protein) and enhanced GSH level (once daily: 31.74 ± 1.035 μM/mg of protein and twice daily: 33.26 ± 0.995 μM/mg of protein) as compared to paclitaxel treated groups (Fig. 4). However, anti-oxidant response with twice application was found to be similar as that of once daily application of formulated dermal gel. Significant observations (One way ANOVA test, P < 0.05) were seen with oral administration of duloxetine hydrochloride (30 mg/kg, once a day for 2 weeks) like reduced TBARS level (2.622 ± 0.12 nM/mg of protein) and elevated GSH level (30.28 ± 0.946 μM/mg of protein) (Fig. 4) in contrast to paclitaxel-treated group. Nevertheless, duloxetine hydrochloride through oral route did not crowd any significant superior signs of anti-oxidant activity in comparison to dermal route.

### Anti-inflammatory activity of formulated dermal gel on sciatic nerve

**Fig. 5:**
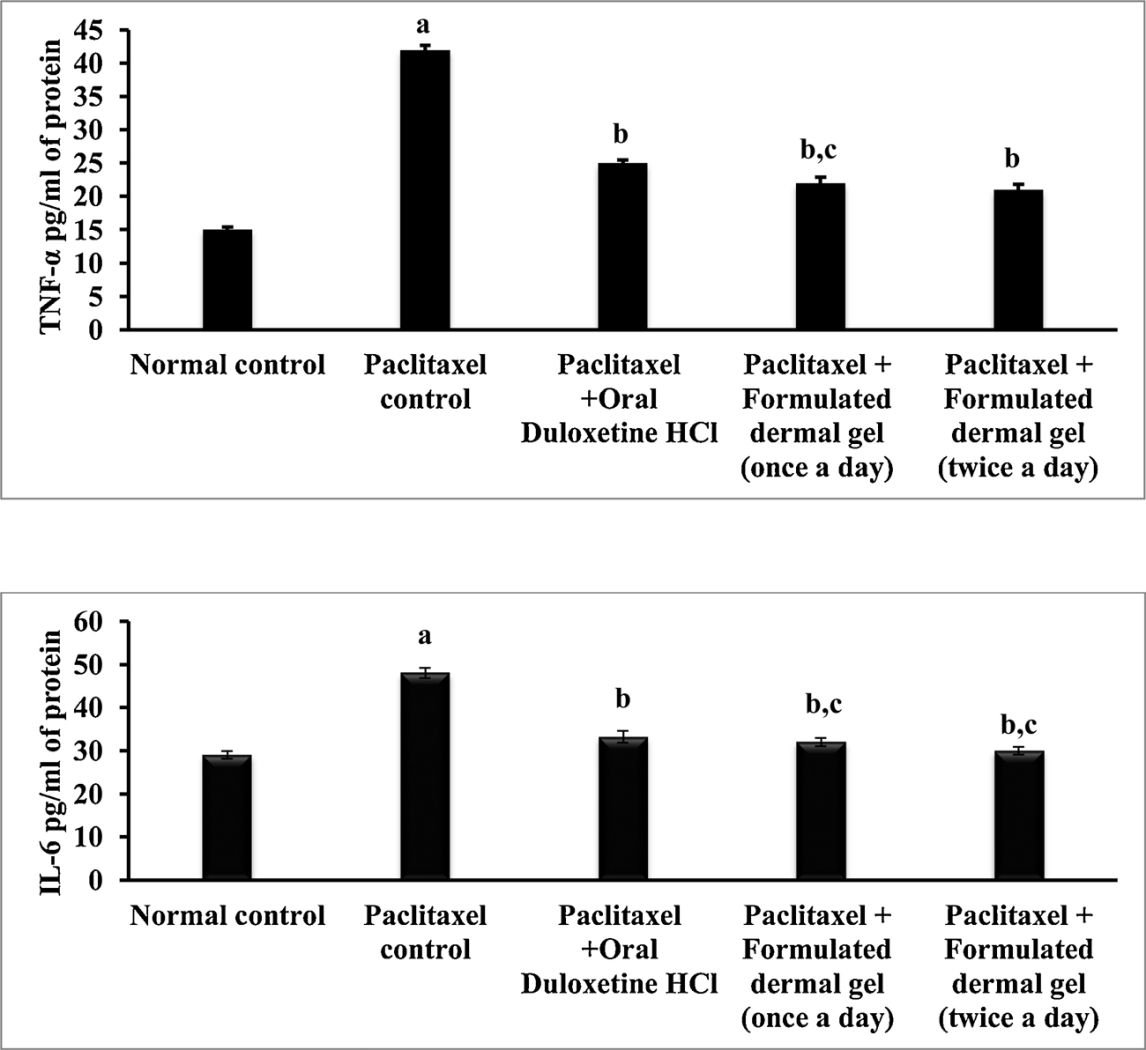
Measurement of TNF-α level (pg/mL of protein) and IL-6 level (pg/mL of protein) in rat sciatic nerve in for normal control, paclitaxel control, Paclitaxel + Oral Duloxetine HCl, Paclitaxel + Formulated dermal gel (once a day), Paclitaxel + Formulated dermal gel (twice a day) groups. **Normal control**: untreated group, **Paclitaxel control**: Paclitaxel (8 mg/kg; *i.p.* in four divided doses) for 4 alternate days, **Paclitaxel + Oral Duloxetine HCl**: Paclitaxel (8 mg/kg; *i.p.* in four divided doses) for 4 alternate days followed by duloxetine HCl (30 mg/kg, once a day for 14 days), **Paclitaxel + Formulated dermal gel (once a day)**: Paclitaxel (8 mg/kg; *i.p.* in four divided doses) for 4 alternate days followed by formulated dermal gel application once a day on hind paws of rats for 14 days, **Paclitaxel + Formulated dermal gel (twice a day)**: Paclitaxel (8 mg/kg; *i.p.* in four divided doses) for 4 alternate days followed by formulated dermal gel application twice a day on hind paws of rats for 14 days. The values are expressed as mean ± standard deviation (SD), n = 6. **a** depicts statistically significant differences (p < 0.05) between paclitaxel control and normal control. **b** depicts statistically significant differences (p < 0.05) between paclitaxel control vs Paclitaxel + Oral Duloxetine HCl/Paclitaxel + Formulated dermal gel (once a day)/Paclitaxel + Formulated dermal gel (twice a day). **c** depicts statistically insignificant differences (p > 0.05) between Paclitaxel + Formulated dermal gel (once a day) vs Paclitaxel + Oral Duloxetine HCl/Paclitaxel + Formulated dermal gel (twice a day).

A significant (Unpaired t test, P < 0.05) rise was observed in levels of TNF-α (42 ± 0.7 pg/mL of protein) and IL-6 (48 ± 1.2 pg/mL of protein) in tissue homogenate of sciatic nerve upon administration of paclitaxel as compared to TNF-α (15 ± 0.4 pg/ml of protein) and IL-6 (29 ± 0.9 pg/mL of protein) levels of normal control animals (Fig. 5).

Once and twice daily application of formulated dermal gel at hind paws of rats for 2 weeks significantly (Two way ANOVA test, P < 0.05) decreased the paclitaxel-induced enhancement of TNF-α (once daily: 22 ± 0.9 pg/ml of protein and twice daily: 21 ± 0.8 pg/ml of protein) and IL-6 (once daily: 32 ± 1.0 pg/ml of protein and twice daily: 30 ± 0.9 pg/ml of protein) levels as compared to paclitaxel-treated group (Fig. 5). However, no significant augmentation was indicated with twice application of formulated dermal gel as compared to once daily application. On the other hand, oral administration of duloxetine hydrochloride (30 mg/kg, once a day for 2 weeks) also in the same way significantly (Two way ANOVA test, P < 0.05) reduced the TNF-α (25 ± 0.5 pg/ml of protein) and IL-6 (33.2 ± 1.4 pg/ml of protein) levels in paclitaxel treated group in comparison to paclitaxel group, however no significant variation was observed when oral group was compared to the formulated gel treated animals (Fig. 5).

### Formulated dermal gel reversed paclitaxel-induced degenerative changes in sciatic nerve histographs along with nerve regenerating signs

#### Hematoxylin and Eosin stained sections

**Fig. 6:**
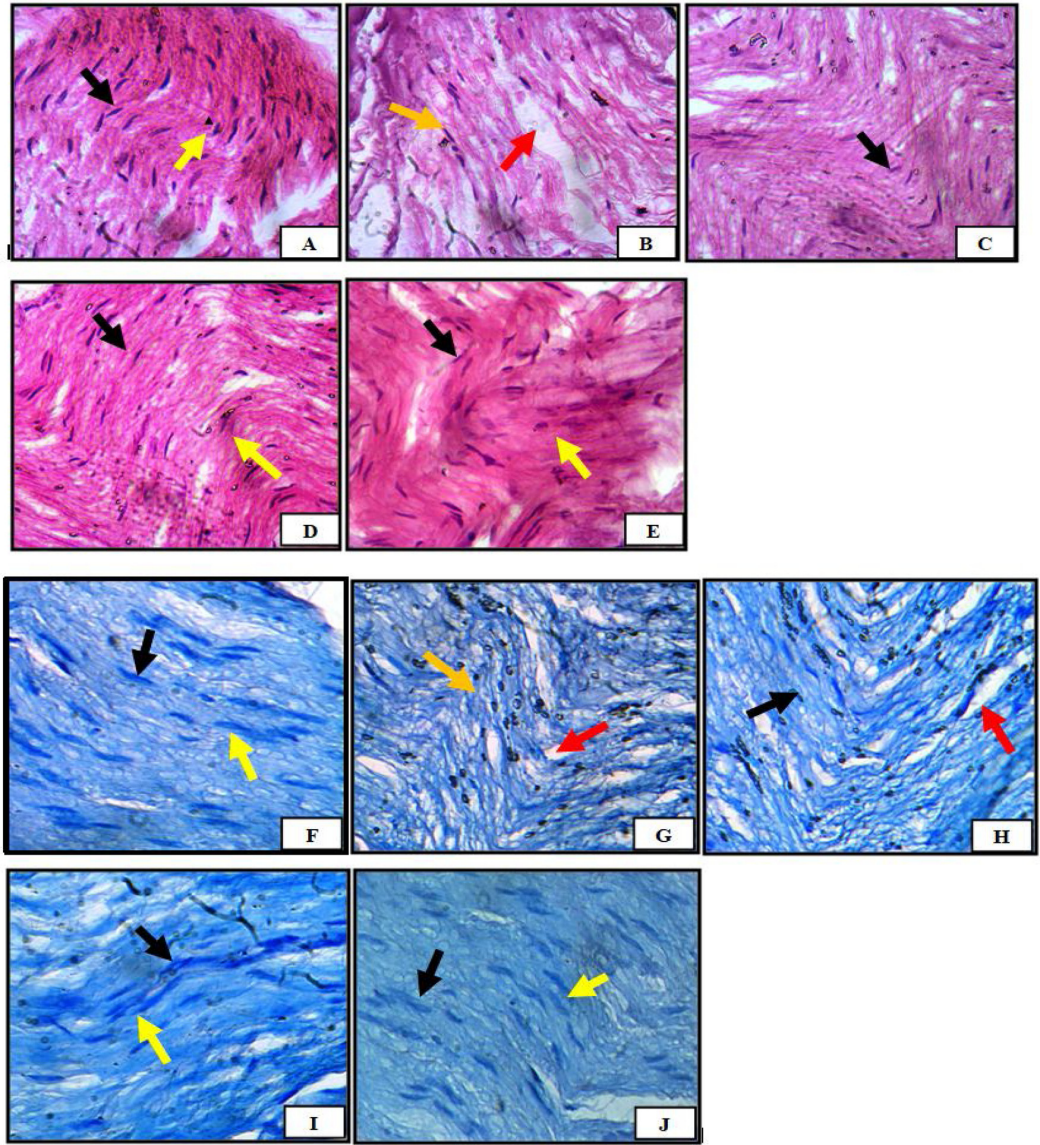
Hematoxylin & eosin and Toluidine blue stained histopathological photomicrographs of the sciatic nerve in for normal control, paclitaxel control, Paclitaxel + Oral Duloxetine HCl, Paclitaxel + Formulated dermal gel (once a day), Paclitaxel + Formulated dermal gel (twice a day) groups. **A and F**: Normal control. **B and G**: Paclitaxel control. **C and H**: Paclitaxel + Oral Duloxetine HCl. **D and I**: Paclitaxel + Formulated dermal gel (once a day). **E and J:** Paclitaxel + Formulated dermal gel (twice a day). Yellow arrow represents- Longitudinally arranged nerve fibres. Black arrow represents- Schwann cell nuclei. Red arrow represents- Necrotic area. Orange arrow represents- Tortuous fibre tract.

To ensure that formulated dermal gel was capable to create normal histology, sciatic nerve sections of paclitaxel (8 mg/kg; *i.p.* in four divided doses) treated animals were exposed to H & E stain. These sections showed marked histopathological changes such as nerve fibre derangement with areas of necrosis and reduction in number of schwann cell nuclei as compared to sections of normal control animals, which bear elongated schwann cell nuclei, longitudinally oriented and arranged axons with myelin sheath. Hind paw application of formulated dermal gel (once and twice a day for 2 weeks) in paclitaxel-treated rats sections illustrated reduced areas of necrosis and derangement of nerve fibres. These sections depicted signs of improvement in terms of orderly arrangement, more schwann cells and partially disintegrated axonal myelin sheath. Moreover, these changes strongly indicated regeneration of otherwise demyelinated nerve fibres, however no significant superiority was observed to reduce paclitaxel-induced degenerative changes in twice topical application in comparison to once daily treatment. Although, sections of oral treatment with duloxetine hydrochloride (30 mg/kg, once a day for 2 weeks) significantly reduced paclitaxel-induced histopathological changes in terms of uniform arrangement of nerve fibres and presence of schwann cells but did not show any dominating and regenerating indication as compared to dermal exposure (Fig. 6).

#### Toluidine blue stained sections

Sciatic nerve sections of paclitaxel-treated animals showed significant disorganization of nerve fibres, weaker staining and more tortuous fibre tracts along with zones of necrosis. Sections from paclitaxel treated animals also showed missing axons, vacuolation and complete demyelination of axons when compared with normal untreated sections. Hind paw application of formulated dermal gel (once and twice a day for 2 weeks) significantly reversed the tortuous fibre tracts into well arranged nerve fibres. Regular, uniform and thickened myelin sheath as well as devacuolation resulted in arrest of necrosis of nerve fibres as compared to sections of paclitaxel group. Conversely, twice application of formulated gel did not produce any extra significant restorative signs which can be compared with once daily treatment. On the other hand, oral delivery of duloxetine hydrochloride resulted in similar responses to that of topical delivery indicated by significant reduction of necrotic areas and restoration of few axonal myelin sheaths as compared to diseased animals (Fig. 6) but no regenerating sign was observed that could be comparable with formulated dermal gel.

## Discussion

In the present study, administration of paclitaxel (8 mg/kg; *i.p.* in four divided doses) for 4 alternate days induced the symptoms of peripheral neuropathy in wistar rats which were confirmed by different behavioral parameters such as mechanical hyperalgesia, cold allodynia, and heat hyperalgesia with significant decrease in paw withdrawal threshold in comparison to normal control animals. The acute symptoms of behavioural alterations started after 24 h of first dose of paclitaxel. The peak values of behavioural alterations were observed after 2 weeks of paclitaxel administration and lasted throughout the experimental study. Statistically significant rise in TBARS and reduction in GSH levels indicated oxidative injury in paclitaxel-treated animals. Moreover, a significant accentuation was also observed in TNF-α and IL-6 levels of paclitaxel treated animals indicating inflammatory insult. Marked histopathological changes such as nerve derangement, axonal swelling, and vacuolar degeneration of nerve fibres were demonstrated in stained sections of sciatic nerve of animals treated with paclitaxel.

Duloxetine hydrochloride is potentially active against various kinds of pain such as fibromyalgic pain, osteoarthritis knee pain and chronic lower back pain. Moreover, it is also the first drug approved by U.S. Food and Drug Administration (FDA) for diabetic peripheral neuropathic pain (Goldstein et al., 2005). However, excessive first pass metabolism, intolerable side effects such as hepatic impairment, renal insufficiency, weight gain, anorexia, fatigue, dry mouth etc and poor nerve repair created a thrust to design an alternate delivery of duloxetine hydrochloride. Hence, to overcome the shortcomings of existing treatment, a duloxetine hydrochloride loaded film forming dermal gel was designed for the management of CIPN. Additionally, contribution of methylcobalamin and geranium oil nourished the formulated dermal gel to promote nerve rejuvenation. Methylcobalamin (active form of VitB_12_) plays a crucial role to preserve the normal functioning of nervous system. It is essential for various the biochemical metabolisms and motor and sensory functions of the nervous system. Furthermore, methylcobalamin promotes synthesis of lecithin that accelerates the myelination in the PNS. Preclinical studies also illustrated the role of methylcobalamin in promoting the peripheral nerve regeneration post injury in rats (Reyes-Garcia et al., 2004). Anti-nociceptive and nerve rejuvenating properties of geranium oil makes it a suitable candidate to enrich the dermal formulation.

Results of the present study indicate that the formulated dermal gel successfully qualified the pharmaceutical characteristics of dermal gel formulations. The formulated dermal gel was pink in color due to presence of methylcobalamin. The developed gel was homogenous with absence of lumps as like marketed formulation. The pH value of formulated dermal gel was identical to normal pH range of the skin as in marketed formulation. Non-newtonian fluid behaviour was followed by the formulated gel. The value of spreadability indicated that the formulated dermal gel can be effortlessly spreadable by little amount of shear. Optimization results have been shown that formulated dermal gel is suitable for topical application.

In the present study, the formulated dermal gel was applied (once daily and twice daily for 2 weeks) on the hind paws of paclitaxel treated rats as scheduled in experimental protocol. it has been observed that application of formlutaed dermal gel to paclitaxel-treated rats significantly improved the behavioral performances, biochemical alterations as well as histopathological changes. The formulated dermal gel may have exerted protective actions by blocking voltage-gated calcium channels to attenuate hyperexcitability, which has been confirmed by significant improvement in the behavioural performances in terms of increase in paw withdrawal threshold. Mitochondrial dysfunction and oxidative stress have been designated as critical indicators of platinum-induced neuropathy. Upon entering neuronal and nonneuronal cells, platins bind to mitochondrial DNA (mDNA) and form mDNA adducts (Di Cesare Mannelli et al., 2012). These pathological products cannot be mended pertaining to lack of DNA repair system in mitochondria (Di Cesare Mannelli, 2013). The destruction of the mitochondrial physiological function leads, in turn, to reduced cellular metabolism. The in vitro studies have suggested that oxaliplatin significantly increases superoxide anion production, induces lipid peroxidation, protein and DNA oxidation in both sciatic nerves and the spinal cord (Waseem et al., 2018). The formulated dermal gel also reduced paclitaxel-induced oxidative injury via promotion of anti-oxidant cascade, as significant decrease in TBARS and rise in reduced GSH levels was indicated. A significant attenuation of paclitaxel-induced inflammatory insult proves the anti-inflammatory activity of formulated gel, which has been confirmed by significant decrease in paclitaxel-induced elevated TNF-α and IL-6 levels in sciatic nerve homogenate. Small fiber neuropathy (SFN) has been associated with many medical conditions including glucose dysmetabolism, connective tissue disease, HIV infection, hereditary diseases, alcoholism, and CIPN (Ghnenis et al., 2018). Nerve conduction studies and electromyography, which are mostly employed for the evaluation of large fiber neuropathies, are normal diagnostic procedures for SFN as well. However, it is difficult to diagnose SFN using these techniques. Hematoxylin and Eosin (H&E) staining is one of the most common stains in histology due to simplicity, reliability and its capacity to portray a wide range of cytoplasmic, nuclear, and extracellular matrix features. H&E principally colours the nuclei of cells dark purple. It also stains schwan cells in particular. Scwann cells are responsible for the production of myelin sheaths in the PNS. Narrow gaps in the myelin sheath between Scwann cells are called nodes of Ranvier. The nodes of Ranvier play a key role during the propagation of action potentials. It provides the typical dark and sharp myelin stain, which greatly facilitates the identification of nerve fibers. Toluidine blue staining on the other hand is a reproducible method for qualitative and quantitative assessments of peripheral nerves, enabling visualization of morphology number of axons and degree of myelination. Arrangement of nerve fibres can be easily visualized with toluidine blue stain.

Furthermore, it has been shown that formulated gel treated sections of sciatic nerve stained with H&E, significantly arrested paclitaxel-induced necrosis and derangement of nerve fibres. Sections of treatment significantly showed orderly arrangement, more schwan cells and partially disintegrated axonal myelin sheath which may be an indication of regeneration of demyelinated nerve fibers. Toluidine blue stained section of sciatic nerve significantly reversed the tortuous fibre tracts into well arranged nerve fibres. Regular, uniform and thickened myelin sheath as well as devacuolation resulted in arrest of necrosis of nerve fibres as compared to sections of paclitaxel treated animals.

Oral administration of duloxetine hydrochloride in paclitaxel-treated rats also results in significant improvement in behavioral performances in terms of increase in paw withdrawal threshold, reduced paclitaxel-induced oxidative stress, inflammation and mildly attenuates the degeneration of nerve fibres. However, dermal delivery of duloxetine hydrochloride contrasted a remarkable superior attenuation of neuropathic symptoms as compared to oral route of drug administration. With the support of the results, the formulated dermal gel is reported to exert battery of valuable effects to combat peripheral neuropathy in terms of anti-nociceptive, anti-oxidant activity, anti-inflammatory activity as well as neuroprotective actions.

## Conclusion

In this investigation, film forming gel was designed to deliver duloxetine hydrochloride through dermal route in a manner to bypass oral route associated crisis. We explored the effectiveness of duloxetine hydrochloride loaded film forming dermal gel against paclitaxel induced peripheral neuropathy. Interestingly, we observed remarkable superior *in-vivo* efficacy with dermal treatment when compared with oral delivery. This could be due to the local administration, increased targetability, sustained drug release of duloxetine hydrochloride and bonus assistance of integrated nourishing supplements (methylcobalamin and geranium oil), which helps in rejuvenation of damaged myelin sheath and promote the growth at axonal level. Formulated dermal gel was noticed to be best-suited dosage form that could be efficacious, cost effective and clinically pertinent option in the effective management of CIPN. Mechanistically, the formulated gel composition described herein is able to combat the peripheral neuropathy due to its anti-nociceptive, anti-oxidant activity, anti-inflammatory activity as well as neuroprotective actions. These outcomes designate the improvement of the condition towards normal stage. Hence, it may be concluded that formulated gel is pharmacologically efficacious for the management of chemotherapy-induced neuropathy and may be excellent therapeutic option for the same.

## Compliance with ethical standards

All animal experiments were carried out in compliance with the CPCSEA (Committee for the Purpose of Control and Supervision of Experiments on Animals) Ministry of Fisheries, Animal Husbandry and Dairying Department of Animal Husbandry and Dairying, Government of India, New Delhi) guidelines.

## Author Contributions

**Jitender Madan** and **Rupinder Sodhi** conceptualized and supervised the study. **Simerjeet Chahal** performed the experiments and prepared the original draft. **Jitender Madan** and **Rupinder Sodhi** reviewed and edited the manuscript.

## Conflicts of Interest

Authors declared no conflict of interest.

## References

[bib0005] Ahmed T.A., Khalid M. (2014). Development of alginate-reinforced chitosan nanoparticles utilizing W/O nanoemulsification/internal crosslinking technique for transdermal delivery of rabeprazole. Life Sci..

[bib0010] Argyriou A.A., Koltzenburg M., Polychronopoulos P., Papapetropoulos S., Kalofonos H.P. (2008). Peripheral nerve damage associated with administration of taxanes in patients with cancer. Crit. Rev. Oncol. Hematol..

[bib0015] Balayssac D., Ferrier J., Descoeur J., Ling B., Pezet D., Eschalier A. (2011). Chemotherapy-induced peripheral neuropathies: from clinical relevance to preclinical evidence. Expert Opin. Drug Saf..

[bib0020] Beutler E., Duron O., Kelly B.M. (1963). Improved method for the determination of blood glutathione. J. Lb. Clin. Med..

[bib0025] Boland B.A., Sherry V., Polomano R.C. (2010). Chemotherapy- induced peripheral neuropathy in cancer survivors. Oncol. Nurse Edn..

[bib0030] Brzezinski K. (2012). Chemotherapy-induced polyneuropathy. Part I. Pathophysiology. Contemp. Onkol..

[bib0035] Carre M., Andre N., Carles G., Borghi H., Brichese L., Briand C., Braguer D. (2002). Tubulin is an inherent component of mitochondrial membranes that interacts with the voltage-dependent anion channel. J. Biol. Chem..

[bib0040] Conklin K.A. (2004). Chemotherapy-associated oxidative stress: impact on chemotherapeutic effectiveness. Integr. Cancer Ther..

[bib0045] Cruccu G. (2007). Treatment of painful neuropathy. Curr. Opin. Neurol. Neurosurg..

[bib0050] Di Cesare Mannelli L., Zanardelli M., Failli P., Ghelardini C. (2012). Oxaliplatin-induced neuropathy: oxidative stress as pathological mechanism. Protective effect of silibinin. J. Pain.

[bib0055] Di Cesare Mannelli L., Zanardelli M., Failli P., Ghelardini C. (2013). Oxaliplatin-induced oxidative stress in nervous system-derived cellular models: could it correlate with in vivo neuropathy?. Free Radic. Biol. Med..

[bib0060] Dougherty P.M., Cata J.P., Cordella J.V., Burton A., Weng H.R. (2004). Taxol-induced sensory disturbance is characterized by preferential impairment of myelinated fiber function in cancer patients. Pain.

[bib0065] Dworkin R.H., O’Connor A.B., Backonja M., Farrar J.T., Finnerup N.B., Jensen T.S., Kalso E.A., Loeser J.D., Miaskowski C., Nurmikko T.J., Portenoy R.K., Rice A.S., Stacey B.R., Treede R.D., Turk D.C., Wallace M.S. (2007). Pharmacologic management of neuropathic pain: evidence-based recommendations. Pain.

[bib0070] Eddy N.B., Touchberry C.F., Lieberman J.E. (1950). Synthetic analgesics: I. Methadone isomers and derivatives. J. Pharmacol. Exp. Ther..

[bib0075] El-Say K.M., Ahmed T.A., Badr-Eldin S.M. (2015). Enhanced permeation parameters of optimized nanostructured simvastatin transdermal films: ex vivo and in vivo evaluation. Pharm. Dev. Technol..

[bib0080] Farguhar-Smith P., Brown M.R.D. (2016). Persistent Pain in Cancer Survivors: Pathogenesis and Treatment Options, in Pain Clinical Updates XXIV (London, UK).

[bib0085] Ferrier J., Pereira V., Busserolles J., Authier N., Balayssac D. (2013). Emerging trends in understanding chemotherapy-induced peripheral neuropathy. Curr. Pain Headache Rep..

[bib0090] Flatters S.J.L., Bennett G.J. (2006). Studies of peripheral sensory nerves in paclitaxel-induced painful peripheral neuropathy: evidence for mitochondrial dysfunction. Pain.

[bib0095] Frank L., Bruce M., Thomas M., Alexander M. (2003). Temporary relief of postherpetic neuralgia pain with topical geranium oil. Am. J. Med..

[bib0100] Ghnenis A.B., Zhaojie R.E., Zhang J., Bushman J.S. (2018). Toluidine blue staining of resin-embedded sections for evaluation of peripheral nerve morphology. J. Vis. Exp..

[bib0105] Goldstein D.J., Lu Y., Detke M.J., Lee T.C., Iyengar S. (2005). Duloxetine vs. Placebo in patients with painful diabetic neuropathy. Pain.

[bib0110] Hershman D.L., Lacchetti C., Dworkin R.H., Lavoie Smith E.M., Bleeker J., Cavaletti G. (2014). Prevention and management of chemotherapy induced peripheral neuropathy in survivors of adult cancers: american society of clinical oncology clinical practice guideline. J. Clin. Oncol..

[bib0115] Hincker A., Frey K., Rao L., Wagner-Johnston N., Ben Abdallah A., Tan B., Amin M., Wildes T., Shah R., Karlsson P., Bakos K., Kosicka K., Kagan L., Haroutounian S. (2019). Somatosensory predictors of response to pregabalin in painful chemotherapy-induced peripheral neuropathy: a randomized, placebo-controlled, crossover study. Pain.

[bib0120] Inoue Y., Furuya K., Matumoto M., Murata I., Kimura M., Kanamoto I. (2012). A comparison of the physicochemical properties and a sensory test of acyclovir creams. Int. J. Pharm..

[bib0125] Izumi Y., Kaji R. (2007). Clinical trials of ultra-high-dose methylcobalamin in ALS. Brain Nerve.

[bib0130] Kamila M.M., Mondal N., Ghosh L.K. (2007). A validated UV Spectrophotometric method for determination of duloxetine hydrochloride. Pharmazie.

[bib0135] Kostich W., Hamman B.D., Li Y.W., Naidu S., Dandapani K., Feng J., Easton A. (2016). Inhibition of AAK1 kinase as a novel therapeutic approach to treat neuropathic pain. J. Pharmacol. Exp. Ther..

[bib0140] Liu Y., Yu X.M., Sun R.J., Pan X.L. (2017). Folate-functionalized lipid nanoemulsion to deliver chemo-radiotherapeutics together for the effective treatment of nasopharyngeal carcinoma. AAPS PharmSciTech.

[bib0145] Lowry O.H., Rosebrough N.J., Farr A.L., Randall R.J. (1951). Protein measurement with the Folin phenol reagent. J. Biol. Chem..

[bib0150] Niehaus W.G., Samuelsson B. (1968). Formation of malonaldehyde from phospholipid arachidonate during microsomal lipid peroxidation. Eur. J. Biochem..

[bib0155] Ozaki K., Hamano H., Matsuura T., Narama I. (2016). Effect of deoxycorticosterone acetate-salt-induced hypertension on diabetic peripheral neuropathy in alloxan-induced diabetic WBN/ Kob rats. J. Toxicol. Pathol..

[bib0160] Pachman D.R., Watsonm J.C., Lustberg M.B., Wagner-Johnston N.D., Chan A., Broadfield L., Cheung Y.T., Steer C., Storey D.J., Chandwani K.D. (2014). Management options for established chemotherapy-induced peripheral neuropathy. Support. Care Cancer.

[bib0165] Polomano R.C., Mannes A.J., Clark U.S., Bennett G.J. (2001). A painful peripheral neuropathy in the rat produced by the chemotherapeutic drug, paclitaxel. Pain.

[bib0170] Randall L.O., Selitto J. (1957). A method for measurement of analgesic activity of inflamed tissue. Arch. Int. Pharmacodyn. Ther..

[bib0175] Reyes-Garcia G., Caram-Salas N.L., Medina-Santillan R., Granados-Soto V. (2004). Oral administration of B vitamins increases the antiallodynic effect of gabapentin in the rat. Proc. West. Pharmacol. Soc..

[bib0180] Rhee Y.S., Chang S.Y., Park C.W., Chi S.C., Park E.S. (2008). Optimization of ibuprofen gel formulations using experimental design technique for enhanced transdermal penetration. Int. J. Pharm..

[bib0185] Rivera E., Cianfrocca M. (2015). Overview of neuropathy associated with taxanes for the treatment of metastatic breast cancer. Cancer Chemother. Pharmacol..

[bib0190] Salvemini D., Little J.W., Doyle T., Neumann W.L. (2011). Roles of reactive oxygen and nitrogen species in pain. Free Radic. Biol. Med..

[bib0195] Schmolka I.R. (1972). Preparation and properties of Pluronic PF-127 gels for the treatment of burns. J. Biomed. Mater. Res..

[bib0200] Sharawy A.M.E., Shukr Marwa Hassan, Elshafeey Ahmed Hassen (2017). Formulation and optimization of duloxetine hydrochloride buccal films: invitro and invivo evaluation. Drug Deliv..

[bib0205] Singh A., Bali A. (2016). Formulation and characterization of transdermal patches for controlled delivery of duloxetine hydrochloride. Anal. Sci. Technol..

[bib0210] Smith E.M.L., Pang H., Cirrincione C., Fleishman S., Paskett E.D., Ahles T., Bressler L.R., Fadul C.E., Knox C., Le-Lindqwister N., Gilman P.B., Shapiro C.L. (2013). Effect of duloxetine on pain, function, and quality of life among patients with chemotherapy-induced painful peripheral neuropathy: a randomized clinical trial. JAMA.

[bib0215] Srivastava N., Patel D.K., Rai V.K., Pal A., Yadav N.P. (2018). Development of emulgel formulation for vaginal candidiasis: pharmaceutical characterization, in vitro and in vivo evaluation. J. Drug Deliv. Sci. Technol..

[bib0220] Staff N.P., Fehrenbacher J.C., Caillaud M., Damaj M.I., Segal R.A., Rieger S. (2019). Pathogenesis of paclitaxel-induced peripheral neuropathy: a current review of in vitro and in vivo findings using rodent and human model systems. Exp. Neurol..

[bib0225] Sudoh Y., Desai S.P., Haderer A.E., Sudoh S., Gerner P., Anthony D.C., De Girolami U., Wang G.K. (2004). Neurologic and histopathologic evaluation after high volume intrathecal amitriptyline. Reg. Anesth. Pain Med..

[bib0230] Sun Y., Lai M.S., Lu C.J. (2005). Effectiveness of vitamin B12 on diabetic neuropathy: systematic review of clinical controlled trials. Acta Neurol. Taiwan..

[bib0235] Thakur V., Prashar B., Arora S. (2012). Formulation and in vitro evaluation of gel for topical delivery of antifungal agent fluconazole using different penetration enhancers. DIT.

[bib0240] Trivedi M.S., Hershman D.L., Crew K.D. (2015). Management of chemotherapy-induced peripheral neuropathy. AJHO.

[bib0245] Velasco R., Bruna J. (2015). Taxane-induced peripheral neuropahty. Toxics.

[bib0250] Waseem M., Kaushik P., Tabassum H., Parvez S. (2018). Role of mitochondrial mechanism in chemotherapy-induced peripheral neuropathy. Curr. Drug Metab..

[bib0255] Wolf S., Barton D., Kottschade L., Grothey A., Loprinzi C. (2008). Chemotherapy-induced peripheral neuropathy: prevention and treatment strategies. Eur. J. Cancer.

[bib0260] Xiao W.H., Bennett G.J. (2012). Effects of mitochondrial poisons on the neuropathic pain produced by the chemotherapeutic agents, paclitaxel and oxaliplatin. Pain.

[bib0265] Zhou M., Gebhart G. (1997). Biphasic modulation of spinal nociceptive transmission from the medullary raphe nuclei in the rat. J. Neurophysiol..

